# The Microbial Capsular Polysaccharide Galactoxylomannan Inhibits IL-17A Production in Circulating T Cells from Rheumatoid Arthritis Patients

**DOI:** 10.1371/journal.pone.0053336

**Published:** 2013-01-08

**Authors:** Eva Pericolini, Alessia Alunno, Elena Gabrielli, Elena Bartoloni, Elio Cenci, Siu-Kei Chow, Giovanni Bistoni, Arturo Casadevall, Roberto Gerli, Anna Vecchiarelli

**Affiliations:** 1 Microbiology Section, Department of Experimental Medicine and Biochemical Science, University of Perugia, Perugia, Italy; 2 Rheumatology Unit, Department of Clinical and Experimental Medicine, University of Perugia, Perugia, Italy; 3 Department of Microbiology and Immunology, Albert Einstein College of Medicine, New York, New York, United States of America; 4 Department of Plastic and Reconstructive Surgery, “La Sapienza” Medical School, University of Rome, Rome, Italy; University Medical Center Freiburg, Germany

## Abstract

The persistence of activated T cells in rheumatoid arthritis (RA) synovium may be attributable to increased homing, increased retention or a possible imbalance between cell proliferation and programmed cell death. Induction of apoptosis may represent a potential therapeutic approach. Galactoxylomannan (GalXM) from the opportunistic fungus *Cryptococcus neoformans* can interact with T cells and induce T-cell apoptosis through the inhibition of CD45 phosphatase activity. The aim of this study was to determine the effect of GalXM on circulating T cells from patients with RA and the underlying mechanisms. GalXM immunomodulating effect on apoptosis and signal transduction pathway involved in IL-17A production was evaluated on T cells. RA T-cell apoptosis, higher than that of control T cells, was further increased by GalXM through induction of caspase-3 activation. Activated T cells expressing the CD45RO molecule and producing IL-17A were the main target of GalXM-induced apoptosis. GalXM induced consistent impairment of IL-17A production and inhibition of STAT3, which was hyperactivated in RA. In conclusion, GalXM triggered apoptosis of activated memory T cells and interfered with IL-17A production in RA. These data suggest therapeutic targeting of deleterious Th17 cells in RA and other autoimmune diseases.

## Introduction

Rheumatoid arthritis (RA) is a chronic autoimmune and inflammatory systemic disease that primarily affects synovial joints. In RA chronically inflamed synovium, a large proportion of the cellular infiltrate consists of CD4^+^ T lymphocytes with a predominance of pro-inflammatory T helper 1 (Th1) and, as recent studies highlight, of Th17 cells on T lymphocytes with counter regulatory activity [1], [2]. Selective inhibition or elimination of these cells is actively being pursued as a potential therapeutic strategy for RA [3], [4], [5]. Since it has been suggested that synovial T-cell activation may be caused by an imbalance between cell proliferation and programmed cell death, another approach of particular interest for the selective depletion of activated T cells is the elicitation of activation-induced cell death [6]. Apoptosis occurs in a variety of physiological situations. The apoptotic stimulus leads to the activation of caspases and/or mitochondrial dysfunction and presents a characteristic pattern of morphological changes [7], [8]. Apoptosis can be triggered through either an extrinsic or an intrinsic pathway. The Fas ligand (FasL)/Fas interaction is the classic initiator of the extrinsic pathway that involves recruitment of FADD (Fas-associated protein with death domain) and subsequent activation of caspase-8. The intrinsic pathway is induced by cellular stress with consequent activation of mitochondria. In some cases the two pathways can synergize and the extrinsic may converge to the intrinsic pathway [9], [10], [11]. The role of Fas and FasL in autoimmune disease is established, as mutations in these proteins can result in proliferative arthritis and lymphadenopathy in murine models and humans [12]. In RA, Fas and FasL have been detected in synovial cells, which are susceptible to Fas-mediated apoptosis induced by an anti-Fas mAb [13]. The inflammatory milieu of the rheumatoid cells is likely to contribute to the degree of Fas-mediated apoptosis, since proinflammatory cytokines such as TNF-α and IL-1β suppress apoptosis *in vitro* [13], [14]. In this context, the possibility to selectively eliminate RA pathogenic synovial T cells by targeted activation of Fas-apoptotic signaling may be of value as novel therapeutic approach [15]. There is much evidence to support a prominent role for Th17 cells in the pathogenesis of human RA [16], [17]. Th17 differentiation is driven by TGF-β, which synergizes with IL-6 to promote IL-23 receptor expression, thus favouring progression toward the Th17 lineage [18]. Th17 are characterized by IL-17A production and high levels of this cytokine have been detected in the synovium of RA patients [19]. In turn, IL-17A induces production of pro-inflammatory mediators such as TNF-α, IL-1β and IL-6 from several joint cells. Even though IL-17A can synergize with these cytokines, it also acts directly by promoting cartilage destruction and bone erosion [16], [17], [18], [19], [20]. In previous papers we demonstrated that a purified polysaccharide from capsular material of the opportunistic fungus *Cryptococcus neoformans* (*C. neoformans*), galactoxylomannan (GalXM), is able to interact directly with T cells via CD45 [21],[22]. The characteristics of this compound have been extensively described [23], [24], [25], [26]. GalXM affects selected immune responses including impairment of T cell proliferation, increase of IFN-γ and IL-10 production, up-regulation of Fas and FasL expression, induction of apoptosis of macrophages and T lymphocytes through activation of caspase-8 [8], [21], [27]. The aim of this study was to determine the immunomodulatory effect of GalXM on peripheral blood T cells from RA patients and the mechanism involved in this process.

## Results

### Effect of GalXM on the Apoptosis of Resting and Stimulated T Cells

Recently, we demonstrated that GalXM is able to directly bind T cells, possibly via CD45, and to generate marked immunoregulation [21], [22].

**Figure 1:**
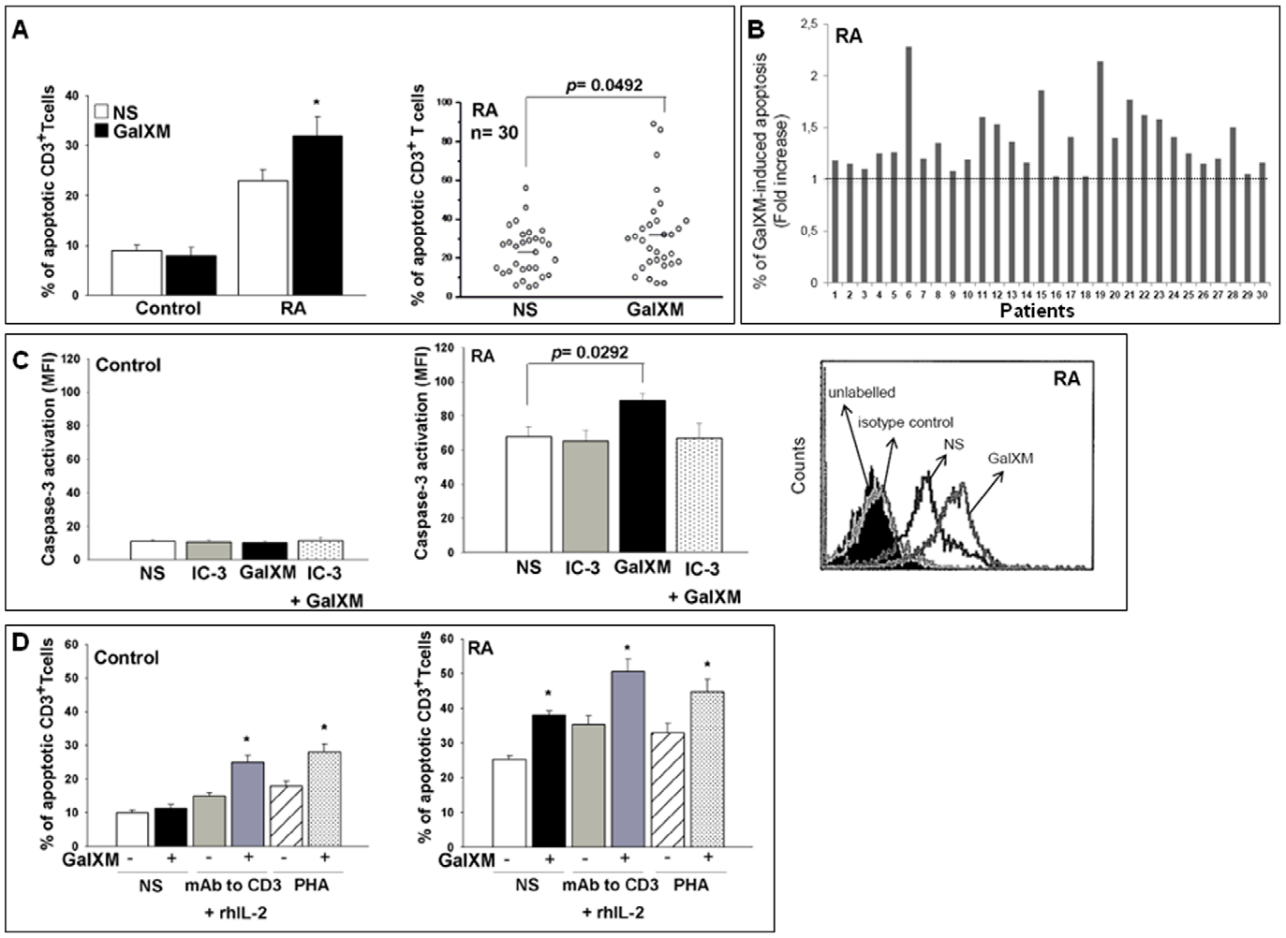
Evaluation of apoptosis. CD3^+^ T cells (1×10^6^/ml) were incubated for 18 h in the presence or absence (unstimulated, NS) of GalXM (10 µg/ml) (A and B), caspase-3 inhibitor (IC-3) (C) or activated for 30 min in the presence or absence (NS) of anti-CD3 mAb (3 µg/ml) and rhIL-2 (20 ng/ml) or PHA (2 µg/ml), and subsequently incubated for 72 h in the presence or absence of GalXM (10 µg/ml) (D). In A, B and D, after incubation, the percentage of cells undergoing apoptosis was evaluated. Data are expressed as percentage of apoptotic CD3^+^ T cells (A and D) *, *p*<0.05 (GalXM treated *vs* untreated cells). In B, the fold increase of percentage of GalXM-induced apoptosis was shown for each RA patient. In C, after incubation, cells were labelled with PE anti-active caspase-3 mAb and analysed using FACScan flow cytometry. Mean ± SEM of MFI of labelled cells is shown as bar graphs and representative FACScan histogram. *p* = 0.0292 (GalXM treated *vs* untreated cells). Error bars denote SEM in all graphs. Panel A and B: Control (n = 10) or RA (n = 30). Panel C: Control and RA (n = 7). Panel D: Control and RA (n = 10).

Firstly, apoptosis of T cells from control and RA patients was evaluated after 18 h of culture in the presence or absence of GalXM. Figure 1A shows that resting T cells from RA patients had a higher level of apoptosis than that observed in control T cells. Although GalXM did not affect apoptosis of T cells from control, it further enhanced the apoptotic process of resting RA T cells (Figure 1A and B). We also evaluated the effect of GalXM on the apoptosis of cells from synovial fluid of osteoarthritis (OA) and RA patients after 18 h of treatment. Our results show that the presence of GalXM increased the percentage of apoptosis in RA cells (17.0±2.1) but not in OA cells (0.4±0.1) compared to untreated cells. We previously demonstrated that GalXM is able to induce apoptosis of T cells via activation of the extrinsic and intrinsic pathways [21]. Therefore, the activation of caspase-3, was evaluated [11], [21]. No modulation of caspase-3 activation was found in T cells from control (Figure 1C). On the contrary, unstimulated T cells from RA patients displayed a consistent activation of caspase-3 and, of note, GalXM-treatment induced a significant further increase of caspase-3 activation (Figure 1C). Caspase-3 inhibitor (IC-3) was able to prevent the GalXM-induced apoptosis enhancement. In subsequent experiments, apoptosis of T cells was evaluated after activation in the presence or absence of anti-CD3 mAb and rhIL-2 or PHA and treatment with GalXM for 72 h, a time chosen on the basis of previous results [22]. The results reported in Figure 1D show that GalXM induces a significant increase of apoptosis in activated T cells from both control and RA patients.

### GalXM Effect on T Cell Proliferation

**Figure 2:**
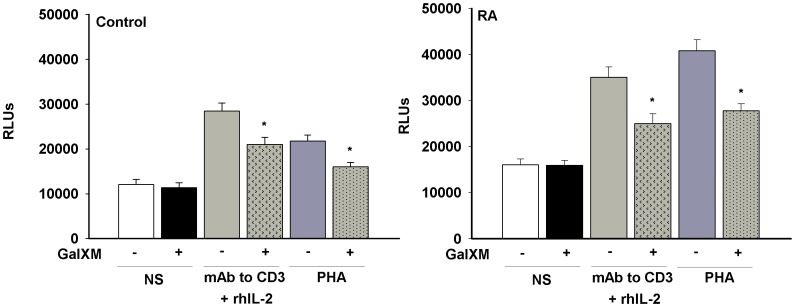
Evaluation of proliferation. CD3^+^ T cells (1×10^6^/ml) were activated for 30 min in the presence or absence (NS) of anti-CD3 mAb (3 µg/ml) and rhIL-2 (20 ng/ml) or PHA (2 µg/ml), washed, and subsequently incubated for 72 h in the presence or absence of GalXM (10 µg/ml). After incubation, cell proliferation was evaluated by ViaLight Plus Kit. *, *p*<0.05 (Control and RA n = 10; GalXM treated *vs* untreated cells). The results reported in the bar graphs are the mean ± SEM.

T cells were activated in the presence or absence of anti-CD3 mAb and rhIL-2 or PHA, and then treated with GalXM. The proliferative response was evaluated after 72 h. Resting RA T cells showed an appreciably higher level of proliferation with respect to that observed from unstimulated control T cells (Figure 2). GalXM treatment did not produce any proliferative changes in unstimulated T cells from control or RA patients, conversely it was able to significantly down-regulate proliferation in activated T cells (Figure 2). The antiproliferative effect of GalXM on T cells from control and RA patients, activated with PHA, was confirmed using carboxyfluorescein succinimidyl ester (CFSE) staining (percentage of inhibition of proliferation in GalXM-treated cells compared to untreated cells; control: 11.1% ±2.4 and RA: 20.1% ±3.7).

This suggests that a state of activation is required for GalXM to exert this effect.

### GalXM Association to the CD45 Molecule on T-cell Surface

Our previous reports suggest that GalXM can be associated with the CD45 molecule of T cell membrane [21], [22].

**Figure 3:**
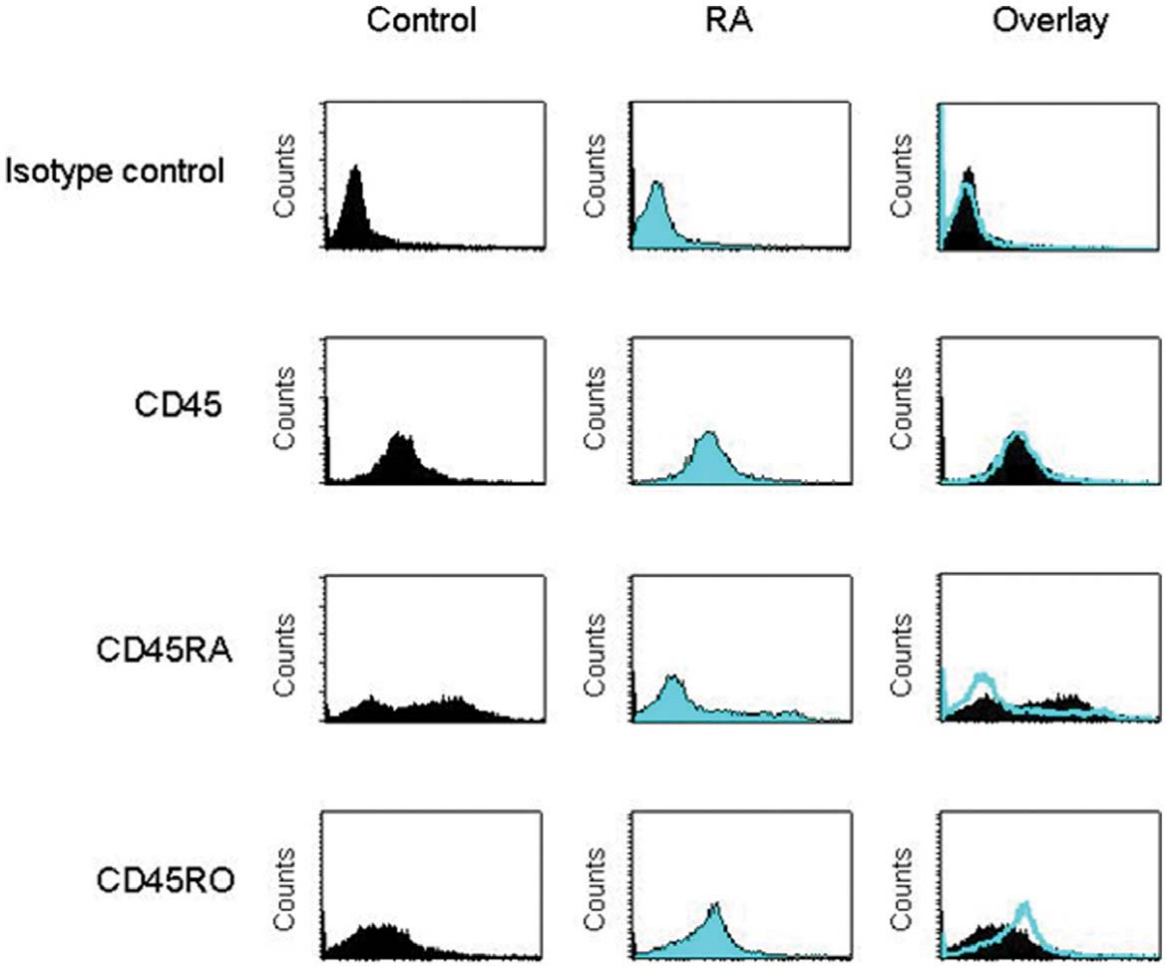
Expression of CD45 isoforms. Purified CD3^+^ T cells (1×10^6^/ml), isolated from control and RA patients, were labelled with PE anti-CD45, anti-CD45RA or anti-CD45RO mAbs and analysed using FACScan flow cytometry. The MFI of labelled cells from one representative experiment out of seven with similar results, were reported (Control and RA n = 7). Background fluorescence values of cells were obtained using isotype control.

**Figure 4:**
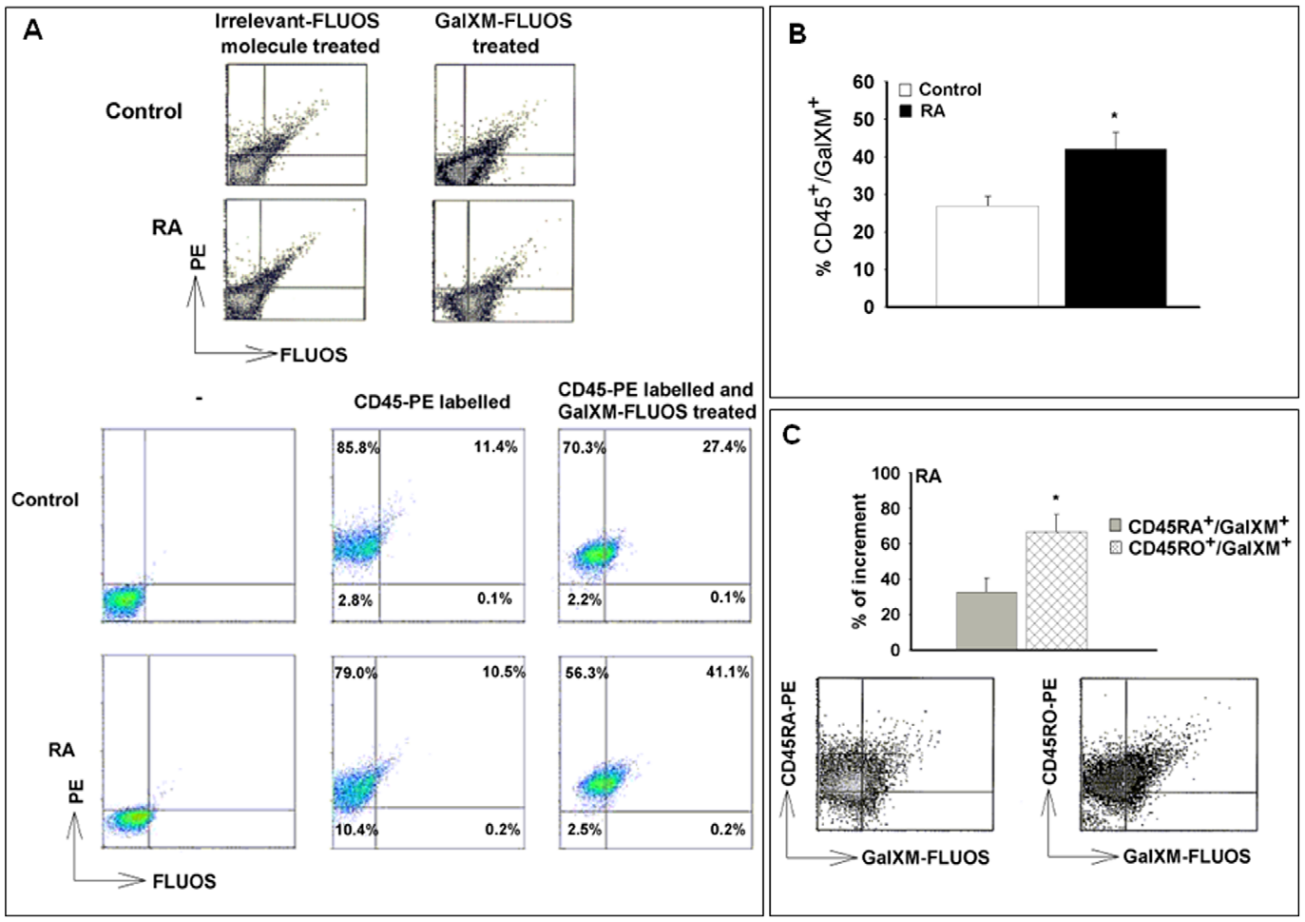
Association between CD45 and GalXM. CD3^+^ T cells (1×10^6^/ml) were incubated for 30 min in the presence or absence of GalXM-FLUOS or an irrelevant-FLUOS molecule (both 10 µg/ml) (A, B and C). After incubation, cells were labelled with PE anti-CD45, anti-CD45RA or anti-CD45RO mAbs and analysed using FACScan flow cytometry. Dot plots of the percentage of double positive cells, from one representative experiment out of seven with similar results, were reported (Control and RA n = 7) (A). In B the percentage of CD45^+^/GalXM^+^ T cells in RA patients compared to control is reported. *, *p*<0.05 (RA *vs* Control, n = 7). In C the relative variation in percentage of CD45RA^+^/GalXM^+^ or CD45RO^+^/GalXM^+^ in relation to double positive CD45^+^/GalXM^+^ in RA patients was calculated. *, *p*<0.01 (CD45RO^+^/GalXM^+^ *vs* CD45^+^/GalXM^+^). The results reported in the bar graphs are the mean ± SEM.

Given that GalXM affects the function of activated T cells, we tested the CD45 isoforms in blood T cells from RA. In our experimental system, there was a consistently higher expression of CD45RA in T cells from control subjects than in cells from RA patients. An increased expression of CD45RO was evident on RA T cell surface (Figure 3). Thus, to test the association between CD45 and GalXM, T cells were treated with GalXM-FLUOS and labelled with PE anti-CD45 mAb. We observed a significant increase in the percentage of double positive CD45^+^/GalXM^+^ T cells after 30 min of GalXM-FLUOS treatment in both control and RA patients (Figure 4A). The association of T cells with GalXM was considerably higher in RA patients than in the control (41.1% and 27.4% respectively).

Naïve T cells switch from high molecular weight of CD45 (CD45RA) to low molecular weight (CD45RO) isoform upon stimulation. The differential expression of CD45 splice variants has frequently been used to distinguish naïve CD45RA^+^ from memory CD45RO^+^ T cells [22], [28].

Moreover, data reported in Figure 4B indicate that the percentage of double positive CD45^+^/GalXM^+^ T cells from RA was significantly incremented as compared to control, and that this increment apparently depended on CD45RO^+^ T cells in RA (Figure 4C). We further analyzed total CD45RO^+^ and total CD45RA^+^ T cells from RA after GalXM treatment and showed that the percentage of the total CD45RO^+^ T cells associated with GalXM was significantly higher than the total CD45RA^+^ T cells (28.5% ±0.4 *vs* 8.8% ±0.5).

Table 1 reports the percentage of GalXM association with CD45 isoforms on T cells from seven RA patients.

**Table 1:** Percentage of GalXM binding to CD45 isoforms on T cells from seven RA patients.

RA patients	CD45RA^+^/GalXM^+^ (%)	CD45RO^+^/GalXM^+^ (%)
RA 1	12	32
RA 2	15	28
RA 3	12	29
RA 4	19	34
RA 5	15	26
RA 6	15	28
RA 7	13	29
Mean ± SEM	14.4±0.8	29.4±0.9*

* *p*<0.01 (Student’s T test), CD45RO^+^/GalXM^+^ *vs* CD45RA^+^/GalXM^+.^

### GalXM Effect on Cytokine Production by T Lymphocytes

**Figure 5:**
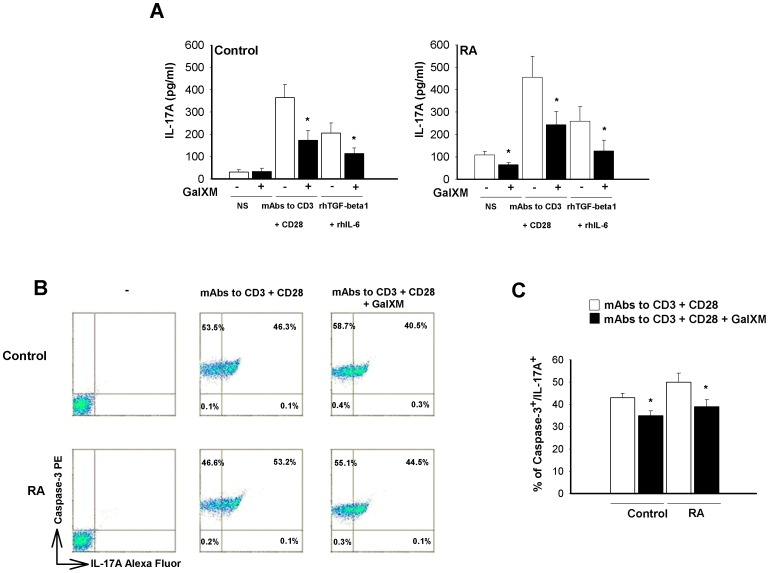
IL-17A production and caspase-3 activation. CD3^+^ T cells (5×10^6^/ml, A or 1×10^6^/ml, B and C) were activated for 30 min in the presence or absence (NS) of soluble anti-CD3 (3 µg/ml) and anti-CD28 (3 µg/ml) mAbs (A, B and C) or rhTGF-β1 (5 ng/ml) and rhIL-6 (20 ng/ml) in mAb to CD3 (B and C) or mAbs to CD3 plus CD28 (A) pre-coated 48-well culture plates, washed, and subsequently incubated for 72 h (A) or 18 h (B and C) in the presence or absence of GalXM (10 µg/ml). In A, after incubation, IL-17A levels were evaluated in culture supernatants by specific ELISA assay. *, *p*<0.05 (Control and RA, n = 7; GalXM treated *vs* untreated cells). In B, after incubation, cells were labelled with PE anti-active caspase-3 and Alexa Fluor anti-IL-17A mAbs and analysed using FACScan flow cytometry. The percentage of double positive cells is shown as dot plots (B) or bar graph (C). *, *p*<0.05 (Control and RA, n = 7; GalXM treated *vs* untreated cells). The results reported in the bar graphs are the mean ± SEM.

Th17 cells represent the major osteoclastogenic Th-cell subset acting by secretion of IL-17A that drives osteoclastogenesis and neoangiogenesis in the RA joint [20], [29], [30], [31], [32]. The majority of IL-17^+^ T cells from RA are CD4^+^ and express mainly the CD45RO isoform as compared to control [33]. IL-17A secretion by RA and control T cells was tested and the effect of GalXM on the production of this cytokine was evaluated. T cells were stimulated in the presence or absence of anti-CD3 and anti-CD28 mAbs or with rhTGF-β1 and rhIL-6, the latter being considered good inducers of Th17 differentiation [34], for 30 min and subsequently treated with GalXM for 72 h. The results show that unstimulated cells from control did not produce IL-17A (Figure 5A). In contrast, an appreciable spontaneous synthesis of IL-17A by resting RA T cells was detected. Of interest, this production was significantly inhibited by treatment with GalXM. In addition, IL-17A production by activated T cells from both control and RA subjects was markedly attenuated after GalXM treatment. We also evaluated IFN-γ production in the same experimental setting. GalXM significantly inhibited IFN-γ production in activated T cells from control (21% of inhibition) and RA subjects (32% of inhibition).

We further explored the possibility that GalXM could induce apoptosis of T cells producing IL-17A. T cells were activated via CD3 and CD28 molecules and subsequently treated with GalXM for 18 h. GalXM treatment of T cells induced a significant decrease in the percentage of active caspase-3^+^ cells producing IL-17A in both RA and control samples (Figure 5B). This effect, however, was more evident in RA (53.2% GalXM-untreated *vs* 44.5% GalXM-treated cells) than in control (46.3% GalXM-untreated *vs* 40.5% GalXM-treated cells). Statistical analysis confirmed this effect (Figure 5C). Indeed, the consistent amount of caspase-3^+^/IL-17A^+^ T cells from control could be due to activation with mAbs to CD3 and CD28 (Figure 5). GalXM treatment seemed to induce an increase of caspase-3 single positive T cells, an increase particularly evident in RA T cells (GalXM-untreated *vs* GalXM-treated cells: 53.5% *vs* 58.7% in control, 46.6% *vs* 55.1% in RA). Actually, this was simply due to the fact that after GalXM treatment there is a shift from double positive caspase-3^+^/IL-17A^+^ cells to single positive caspase-3^+^ cells. However, GalXM did induce an increase of the mean of fluorescence intensity (MFI) of caspase-3 in double positive caspase-3^+^/IL-17^+^ activated T cells from the control and RA patients (control: 63.7±2.8 to 79.5±3.9 and RA: 63.2±6.5 to 77.8±6.5).

### GalXM Effect on STAT3 Expression in T Lymphocytes

**Figure 6:**
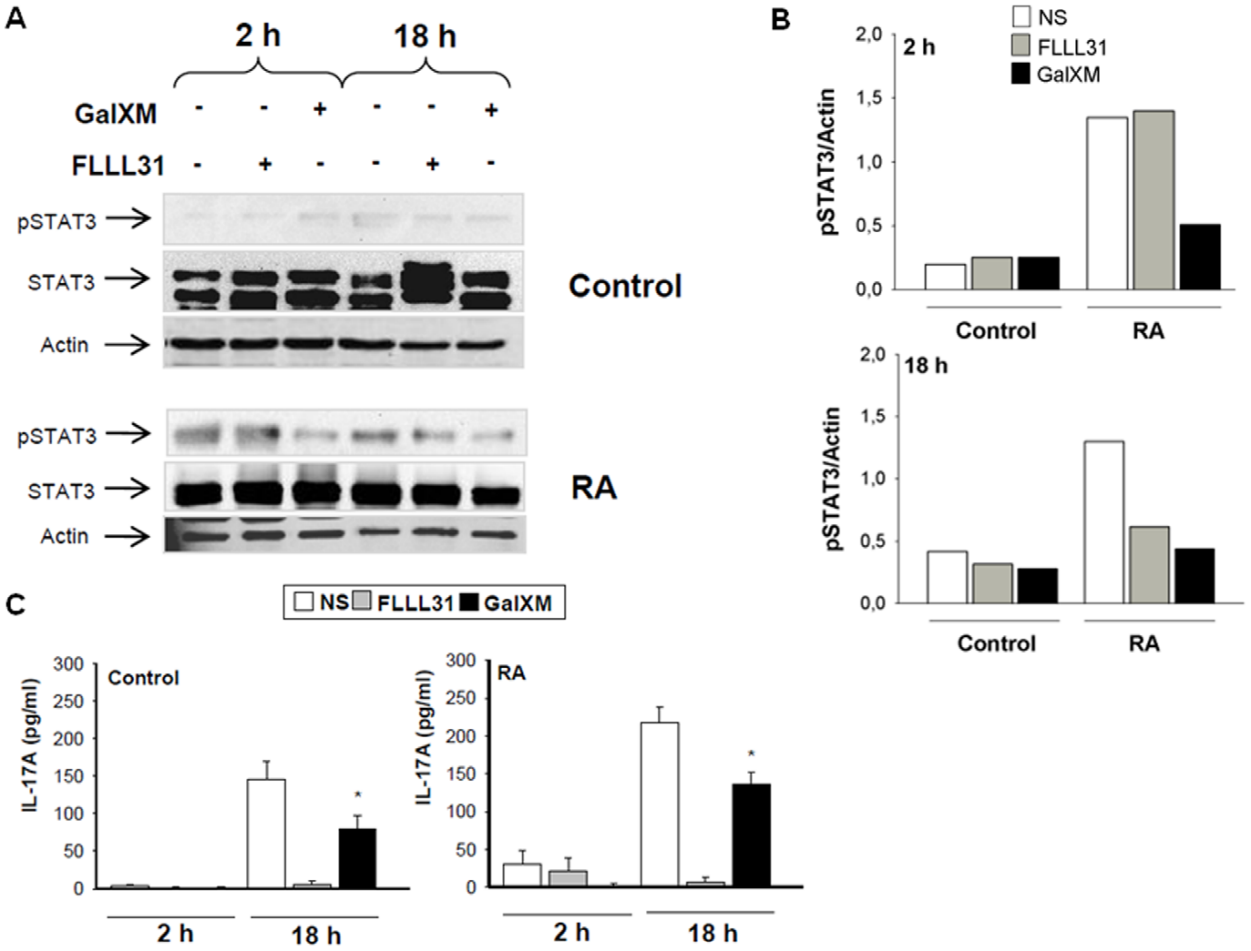
Phospho-STAT3 activation and IL-17A production. PBL (5×10^6^/ml) were activated for 30 min with soluble anti-CD3 (3 µg/ml) and anti-CD28 (3 µg/ml) mAbs, washed, and subsequently incubated for 2 or 18 h in the presence or absence of GalXM (10 µg/ml) or FLLL31 (5 µmol/L). After incubation, cell lysates were analysed by Western blotting. Membranes were incubated with anti-pSTAT3 and anti-STAT3 Abs. Actin was used as an internal loading control (A). Normalization was shown in panel B. Culture supernatants were collected to test IL-17A levels by specific ELISA assay (C). *, *p*<0.05 (Control and RA, n = 7; GalXM treated *vs* untreated cells). The results reported in the bar graph of panel C are the mean ± SEM.

**Figure 7:**
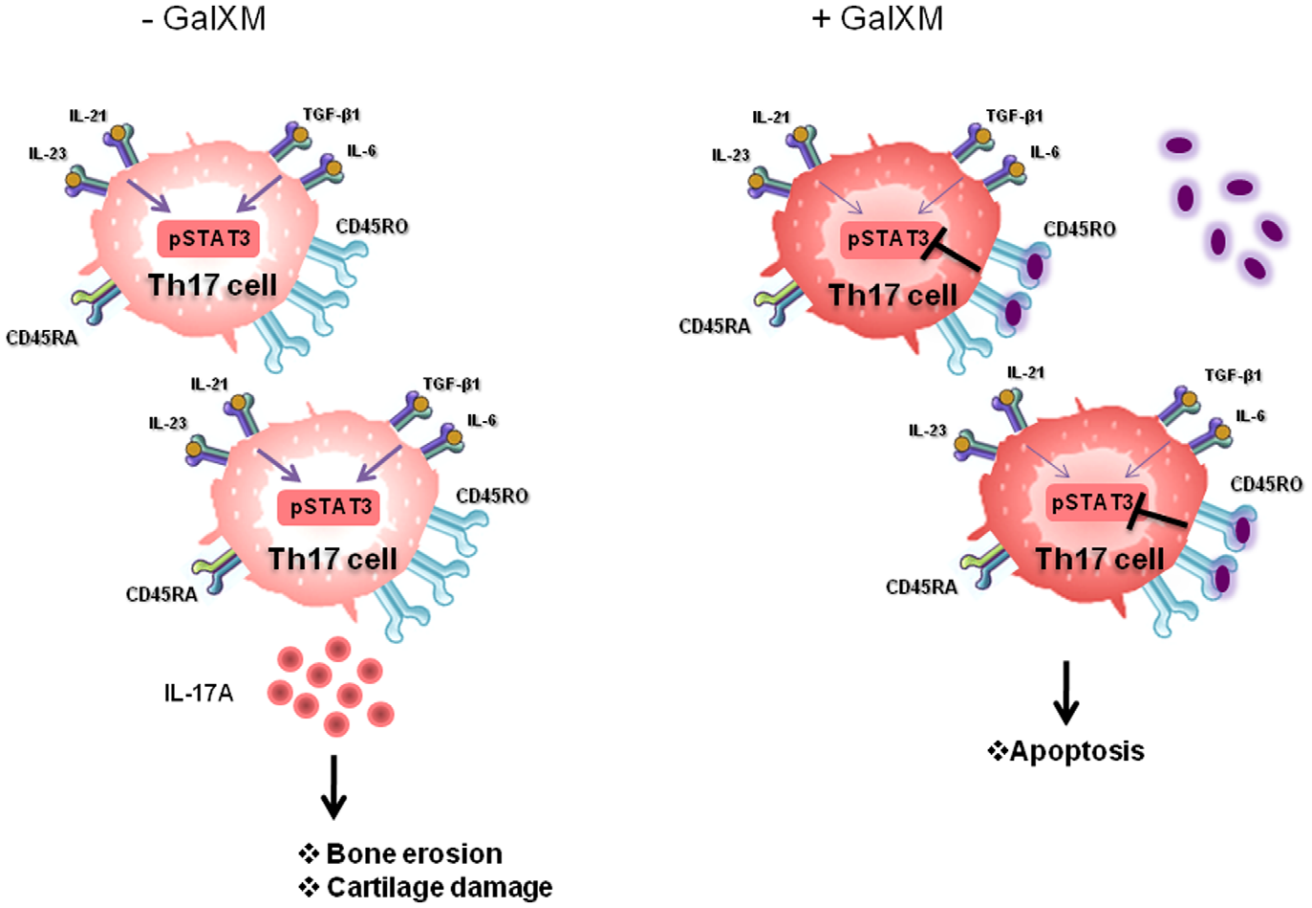
Schematic representation of GalXM effect in T cells from RA patients. RA differentiated Th17 cells express high levels of CD45RO and produce IL-17A through pSTAT3 activation, causing cartilage destruction and bone erosion. GalXM interacts with Th17 cells by selectively binding to CD45RO. This interaction leads to the inhibition of pSTAT3 activation, IL-17A production and apoptosis induction.

STAT3 is a master regulator of Th17 cells, analogous to STAT4 and STAT1 in Th1 cells and STAT6 in Th2 cells [35], [36]. Therefore, we analyzed the possibility that GalXM affected STAT3 expression in T cells. FLLL31 was used as a negative control of phospho-STAT3 activation and IL-17A production (Figure 6A, B and C). Our results show that GalXM was able to inhibit phospho-STAT3 activation of RA peripheral blood lymphocytes (PBL) activated by anti-CD3 and anti-CD28 mAbs after 2 and 18 h of treatment (Figure 6A and B). Moreover, GalXM was also able to significantly inhibit IL-17A production by activated PBL from RA patients after 18 h (Figure 6C). In control PBL treated as above described, phospho-STAT3 was activated after 18 h, and this activation was inhibited, as well as IL-17A production, by GalXM-treatment (Figure 6A, B and C). The proposed mechanism for the GalXM-induced effect is reported in Figure 7.

## Discussion

A general consensus exists about the central role of T cells, and in particular CD4^+^, in initiating and modulating RA immune pathogenesis via the recognition of some unknown antigens [1]. In fact, the abundance of activated CD4^+^ T cells in the synovium of individuals with RA, especially people who express certain allelic forms of the MHC II β-chain (e.g., HLA-DRB1*0401), indicates that the process of antigen recognition by CD4^+^ cells plays a critical role in the pathogenesis of RA [1]. In this context, the observation that autoAbs associated with RA (e.g. against citrullinated proteins) have undergone T-cell dependent isotype switching and somatic hypermutation can also be taken as evidence for the contribution of CD4^+^ cells to the pathogenic mechanisms of the disease [1]. Moreover, CD4^+^ T cells appear to be also involved in some extra-articular manifestations of RA [37]. Finally, the ability of the CD28 molecule antagonist and CD4^+^ T cell immunosuppressant Abatacept to achieve disease remission in RA patients further supports the notion that T cells are actively involved in RA pathogenesis. Several problems, however, are documented about trying to eliminate T cells. RA disease remission is achievable only in a subset of patients. In addition, early pharmacodynamic studies revealed that the beneficial effect of T-cell depletion could be lost because of the preferential killing of naïve T cells, rather than pathogenic T cells [38]. Therefore, therapies specifically targeting pathogenic T cells may lead to clinical benefits in RA. Accordingly, it has been reported that depletion of specific collagen-reactive T cells ameliorated collagen-induced arthritis without systemic immunosuppression and, importantly, osteoclastogenesis was inhibited by apoptotic induction of activated CD4^+^ T cells using anti-Fas mAb [5], [39].

Our recent data showed that a microbial polysaccharide, GalXM, dampens T cell activation in terms of inhibition of proliferation and apoptosis induction [8], [21], [22]. In the present study, we demonstrated that i) peripheral blood T cells from RA patients showed an appreciable level of apoptosis, which was significantly increased upon stimulation with GalXM; ii) the observed apoptosis was related to GalXM-mediated induction of caspase-3 activation; iii) the major target population of GalXM-induced apoptosis was activated T cells producing IL-17A; iv) treatment with GalXM leads to significant impairment of IL-17A production; v) the GalXM-mediated effects include inhibition of STAT3, which is hyperactivated in RA.

We previously demonstrated that GalXM induced apoptosis of activated T cells [8], [21], [22] via interaction with glycoreceptors and in particular with CD45 [21], [22]. Given that the majority of leukocytes express CD45 [28], the possibility arises that GalXM could influence the development of the immune response in other ways. In the present report we demonstrate that T cells of RA patients are highly susceptible to the inhibitory effects of GalXM, including decrease of proliferation as well as induction of apoptosis via caspase-3 activation. Importantly, the inhibitory activity was essentially exerted on activated T cells. Given that unstimulated RA T cells displayed an appreciable basal level of activation compared to control T cells, it is not surprising that GalXM inhibitory effect was particularly evident in RA T cells. Indeed, unstimulated T cells from RA patients, unlike controls, showed appreciable levels of proliferation, apoptosis, cytokine production and CD45RO expression [33]. Despite T cells isolated from controls and RA patients expressed similar levels of CD45, T cells from controls showed significantly higher levels of CD45RA than those from RA patients, while CD45RO was predominantly expressed on RA T cells.

CD45 is a tyrosine phosphatase essential for antigen receptor-mediated signal transduction, regulating Src family members that initiate TCR signaling. CD45 is being attributed a new emerging role as an apoptosis regulator [21], [22]. Cross-linking of the extracellular portion of the CD45 by mAbs and by galectin-1 can induce apoptosis in T and B cells [21], [40]. Multiple isoforms of CD45 exist as a result of the alternative RNA splicing of the exons 4, 5 and 6 (designated A, B and C). For example, naïve T cells express the larger CD45RA isoform while activated and memory T cells carry the smaller CD45RO isoform [41]. Recent findings suggest that the CD45RO population predominates in the peripheral blood and even more in the synovial fluid of patients with RA [33]. That GalXM tends to be preferentially associated with T cells expressing CD45RO isoform and induces apoptosis in these cells involved in the pathogenesis of RA, is therefore an important observation. Moreover, it has been observed that the presence of CD45RO^+^ T cells is maintained by an impairment of their apoptosis in RA.

Interleukin-17 plays a critical role in the pathogenesis of RA. IL-17A is able to foster inflammation by inducing a variety of proinflammatory mediators, including cytokines, chemokines and other mediators of bone and cartilage destruction in synovial fibroblasts, monocytes, macrophages and chondrocytes. RA synovial explants spontaneously produce IL-17A, and increased levels of IL- 17A are found in RA synovial fluid compared with osteoarthritis synovial fluid [42]. IL-17A has previously been identified in T lymphocytes from rheumatoid synovial tissue, especially CD4^+^CD45RO^+^ T cells. These observations support a role for Th17 cells in RA. In this line, recent data strongly suggest that Th17 cells are key effector cells in driving the transition from acute to chronic phase of RA inflammation [42]. In addition, the critical role of Th17 cells has been highlighted by the fact that, in synovitis associated with RA, Th17 cells are involved in driving the active acute phases [42]. It is well known that Th1 also plays an important role in the pathogenesis of RA [43]. The inhibitory effect of GalXM was evident in Th1 cells as suppression of IFN-γ production by these cells.

In our experimental system, we demonstrated that peripheral T cells from RA spontaneously produced appreciable amounts of IL-17A, suggesting that Th17 cells are present in the peripheral blood. To our knowledge this is the first demonstration of spontaneous secretion of IL-17A from peripheral T cells from RA patients. This observation is particularly relevant given that lymphocyte infiltration is mainly perivascular with a T-cell predominance [1].

Even though the most prominent immunopathologic events develop in situ in the joints, immune alterations are not limited to local clonal expansion of synovium-infiltrating lymphocytes but also involve the majority of circulating T cells. In this context, peripheral blood T cells play a critical role and the inhibitory effect of GalXM should be of particular interest.

It has been suggested that peripheral blood mononuclear cells (PBMC) are highly activated in RA, and this observation is supported by a strong surface expression of CD45RO, as well as CD40L, CD69, CD25, HLA-DR, CD39 and CD28 molecules on T cells [44]. Given that Th17 cells are predominantly CD45RO^+^, our data do suggest that GalXM is associated preferentially with activated T cells, in particular with CD45RO^+^ cells. This implies that GalXM particularly targets Th17 cell expansion and this theory is supported by the observation that GalXM inhibits IL-17A production and STAT3 phosphorylation.

Indeed, the inhibitory effects of GalXM are closely related to those of methotrexate, which is considered the gold standard for the treatment of RA, acting through induction of apoptosis and inhibition of cell proliferation [45]. As a consequence, GalXM could be considered as a novel therapeutic option for supporting or substituting the current pharmacological treatment of RA. Moreover, GalXM treatment could also imply inhibition of Th17 cell generation and proinflammatory cytokine production, pivotal events leading to chronic inflammation. These data may help to pursue therapeutic targeting of deleterious Th17 cells in RA disease [16].

## Materials and Methods

### Cryptococcal Polysaccharide and Other Reagents

GalXM was purified as described elsewhere [40], [46]. A fluorescein derivative of GalXM (GalXM-FLUOS) was prepared by incubating GalXM with [5-(4,6-Dicholotriazinyl) aminofluorescein] (5-DTAF, Chem Progress s.r.l., Sesto Ulteriano, MI, Italy) [22], [47]. All reagent and media were negative for endotoxin, as assessed by Limulus amebocyte lysate assay (QCL-1000, BioWhittaker).

### Patients

Sixty-eight consecutive patients meeting the American College of Rheumatology classification criteria for RA and followed up at the Rheumatology Unit of the University of Perugia, were included in the study [48]. This cohort included 53 women and 15 men with a mean age of 62±9 (mean ± SD) and disease duration of 100±86 months. Among patients, 79% and 69% were rheumatoid factor and anti-CCP antibody positive, respectively. The mean Disease Activity Score, as evaluated including 28 swollen and tender joint count (DAS28), was 3.2±1.2 at the time of sample collection. At enrolment, 25 patients were receiving traditional disease-modifying anti-rheumatic drugs in monotherapy (methotrexate, sulphasalazine, hydroxychloroquine) and 42 were taking anti-tumor necrosis factor-α blockers (14 in monotherapy and 28 in combination with methotrexate). Twenty-one patients were also receiving low doses of corticosteroids (5 mg/day of prednisone or equivalent). Thirty-four healthy subjects matched for age and sex acted as normal control (NC, control). Informed consent was obtained from all subjects prior to sample collection. Local Ethical Committee approval was received for the study.

### Cell Separation

Heparinized venous blood was obtained from control and RA patients. Synovial fluids were obtained from 3 patients with RA and 3 patients with osteoarthritis (OA). PBMC were separated by density gradient centrifugation on Ficoll-Hypaque (Pharmacia). PBMC were reacted with anti-human CD3 mAb-conjugated MicroBeads (Miltenyi Biotec) and CD3^+^ T lymphocytes were purified by magnetic separation. In selected experiments, PBMC were plated on culture flasks for 1 h in RPMI 1640 at 37°C and 5% CO_2_. After incubation, the non adherent cellular fraction, PBL, was recovered.

### Evaluation of Apoptosis

CD3^+^ T cells from blood samples of control and RA patients and from synovial fluid of OA and RA patients (1×10^6^/ml) were incubated for 18 h in the presence or absence of GalXM (10 µg/ml) in RPMI 1640 with L-glutamine plus 10% FCS (complete medium) (all from Gibco BRL, Paisley, Scotland) at 37°C and 5% CO_2_. In selected experiments, a 48-well culture plate was pre-coated with anti-CD3 mAb (IgG_1k_ isotype; 2 µg/ml) (ImmunoTools GmbH, Friesoyte, Germany). CD3^+^ T cells (1×10^6^/ml) were seeded in mAb to CD3 (2 µg/ml) pre-coated 48-well culture plates and activated for 30 min in the presence or absence of soluble anti-CD3 mAb (3 µg/ml) and rhIL-2 (20 ng/ml) (ImmunoTools) or PHA (2 µg/ml) (Sigma-Aldrich, St. Louis, MO) in complete medium at 37°C and 5% CO_2_. After activation, cells were washed and then incubated for 72 h in the presence or absence of GalXM (10 µg/ml) in complete medium. After stimulation, cells were centrifuged, resuspended in hypotonic propidium iodide (PI) solution (50 µg/ml) (Sigma-Aldrich) and kept for 1 h at room temperature (RT). The percentage of cells undergoing apoptosis was evaluated by flow cytometry analysis as previously described [49]. Data are expressed as percentage of apoptotic cells.

### Flow Cytometry Analysis

CD3^+^ T cells (1×10^6^/ml) were incubated for 18 h in the presence or absence of GalXM (10 µg/ml) or IC-3 (dilution 1/1000) (Z-DEVD-FMK, Vinci Biochem, Vinci, FI, Italy) in complete medium at 37°C and 5% CO_2_. After incubation, cells were fixed with 1.5% formalin for 10 min at RT, washed, incubated with absolute methanol (500 µl/10^6^cells) for 10 min on ice and incubated with PE-conjugated anti-active caspase-3 mAb (IgG isotype; 20 µl/tube) (BD Biosciences, San Jose, CA) for 20 min on ice. After incubation, cells were washed, resuspended in 0.5 ml of fluorescence buffer (FB) and analyzed by flow cytometry. Data are expressed as MFI of labelled cells. In selected experiments, CD3^+^ T cells (1×10^6^/ml) were incubated for 30 min in the presence or absence of GalXM-FLUOS or an irrelevant-FLUOS synthetic decapeptide (irrelevant-FLUOS molecule), previously proven to be devoid of activity *in vitro* [50], (both 10 µg/ml) as above described. After incubation, cells were fixed with 1.5% formalin as above, washed and reacted with PE-conjugated anti-CD45 (IgG_1_ isotype; 2 µl/tube) (ImmunoTools), anti-CD45RA (IgG_2b_ isotype; 1 µl/tube) (Miltenyi Biotec) or anti-CD45RO (IgG_2a_ isotype; 5 µl/tube) (BD Bioscience) mAbs for 40 min on ice. After incubation, cells were washed, resuspended in 0.5 ml of FB and analyzed by two-color flow cytometry. Results are shown as dot plot of the percentage of double positive cells and as FACScan histograms of the MFI of single positive T cells. Results are from one representative experiment out of seven with similar results. The relative variation in the percentages of CD45RA^+^/GalXM^+^ and CD45RO^+^/GalXM^+^ was calculated for double positive CD45^+^/GalXM^+^ T cells from RA patients. We also selected total CD45RO^+^ or total CD45RA^+^ cells after GalXM treatment and then calculated the percentage of the total CD45RO^+^ T cells associated with GalXM in comparison to the total CD45RA^+^ T cells. Purified CD3^+^ T cells (1×10^6^/ml) were seeded in mAb to CD3 (2 µg/ml) pre-coated 48-well culture plates and activated for 30 min in the presence or absence of soluble anti-CD3 (3 µg/ml) and anti-CD28 (IgG_1_ isotype; 3 µg/ml) (ImmunoTools) mAbs in complete medium. After activation, cells were washed and then incubated for 18 h in the presence or absence of GalXM (10 µg/ml) in complete medium. Brefeldin A (10 µg/ml) was added 2 h after GalXM stimulation [51]. After incubation, cells were washed, fixed with 1.5% formalin for 10 min at RT, incubated with absolute methanol (500 µl/10^6^cells) for 10 min on ice, washed and incubated with Alexa Fluor-conjugated anti-IL-17A mAb (IgG_1k_ isotype; 5 µl/test) (BD Bioscience) for 20 min on ice. Afterwards, the cells were washed and incubated with PE-conjugated anti-active caspase-3 mAb (20 µl/tube) for 20 min on ice. Cells were then washed, resuspended in 0.5 ml of FB and analysed by two-color flow cytometry. Representative dot plots show the percentage of double positive cells for caspase-3 and IL-17A. Dot plots were obtained acquiring only purified CD3^+^ T cells from controls and RA patients. Data are from one representative experiment out of seven with similar results. Autofluorescence was assessed using untreated cells. Control staining of cells with irrelevant antibody was used to obtain background fluorescence values.

### Evaluation of Proliferation

CD3^+^ T cells (1×10^6^/ml) were seeded in mAb to CD3 (2 µg/ml) pre-coated 96-well culture plates and activated for 30 min in the presence or absence of soluble anti-CD3 mAb (3 µg/ml) and rhIL-2 (20 ng/ml) or PHA (2 µg/ml) in complete medium. Cells were washed and then incubated for 72 h in the presence or absence of GalXM (10 µg/ml) in complete medium. After stimulation, cell proliferation was evaluated by an ATP bioluminescence kit (Via Light kit, Lonza Rockland Inc., ME). Luminescence was measured by a luminometer (Infinite 200, Tecan Group Ltd, Männedorf, Switzerland). In selected experiments, freshly prepared CFSE (Molecular Probes, Eugene, OR, USA) was added to a final concentration of 10 µm and the cells were incubated for 10 min at 37°C. Excess CFSE was quenched by adding 10 volumes of ice-cold RPMI 1640 medium containing 10% FCS and incubating the cells for 5 min on ice. CFSE-labeled cells were then washed three times with RPMI 1640 medium containing 10% FCS and cultured with the indicated stimuli. After stimulation, cell proliferation was evaluated by flow cytometry analysis.

### Cytokine Production

CD3^+^ T cells (5×10^6^/ml) were seeded in mAbs to CD3 plus CD28 (both 2 µg/ml) pre-coated 48-well culture plates and activated for 30 min in the presence or absence of soluble anti-CD3 (3 µg/ml) and anti-CD28 (3 µg/ml) mAbs or rhTGF-β1 (5 ng/ml) (PeproTech GmbH, Hamburg, Germany) and rhIL-6 (20 ng/ml) (ImmunoTools) in complete medium. After activation, cells were washed and then incubated for 72 h in the presence or absence of GalXM (10 µg/ml) as above described. The supernatants were collected and tested for IL-17A or IFN-γ levels by specific ELISA assay (Biosource, Camarillo, CA). PBL (5×10^6^/ml) were seeded in mAb to CD3 (2 µg/ml) pre-coated 48-well culture plates and activated for 30 min in the presence of soluble anti-CD3 (3 µg/ml) and anti-CD28 (3 µg/ml) mAbs in complete medium. Cells were washed and then incubated for 2 h or 18 h in the presence or absence of GalXM (10 µg/ml) or with the inhibitor of STAT3 phosphorylation (FLLL31) (5 µmol/L) (Sigma-Aldrich) as above described [52]. The supernatants were collected and tested for IL-17A levels by specific ELISA assay (Biosource). Cytokine titers were calculated relative to standard curves.

### Western Blot Analysis

PBL (5×10^6^/ml) were seeded in mAb to CD3 (2 µg/ml) pre-coated 48-well culture plates and activated for 30 min in the presence of soluble anti-CD3 (3 µg/ml) and anti-CD28 (3 µg/ml) mAbs in complete medium. Cells were washed and then incubated for 2 or 18 h in the presence or absence of GalXM (10 µg/ml) or FLLL31 (5 µmol/L) as previously described [52]. After incubation, cells were washed and lysed with mammalian protein extraction reagent in the presence of protease and phosphatase inhibitors (all from Pierce, Rockford, IL). Protein concentrations were determined with a BCA protein Assay Reagent kit (Pierce). The lysates (30 µg) were separated by sodium dodecyl-sulfate-10% PAGE, transferred to a nitrocellulose membrane (Pierce) for 1 h at 100 V in a blotting system (Bio-Rad, Hercules, CA). The membranes were incubated overnight with goat polyclonal anti-phospho-STAT3 (Tyr 705) Ab (dilution 1/200) (Santa Cruz Biotechnology, Delaware Avenue, Santa Cruz, CA) in blocking buffer. Detection was achieved with the appropriate secondary Ab coupled to HRP, followed by Chemilucent Trial Kit (Chemicon Int., Temecula, CA). Immunoblotting with rabbit polyclonal anti-STAT3 and anti-Actin Abs (both diluted 1/200) (both from Santa Cruz) were used as internal loading controls. Immunoreactive bands were visualized and quantified by Chemidoc Instrument (Bio-Rad Laboratories, Hercules, CA). In particular, a quantitative analysis of the region of interest was performed as previously described [53].

### Statistical Analysis

The results reported in the bar graphs are the mean ± SEM from duplicate samples of 7–10 separate experiments. Student’s t test was used for statistical analysis, except for proliferation in which the statistic was calculated according to Mann-Whitney U-test. A value of *p*<0.05 was considered significant.
